# The Molecular Pathology of Non-Malignant Haematological Disease

**DOI:** 10.3389/bjbs.2026.14743

**Published:** 2026-05-22

**Authors:** A. D. Blann, R. G. Dunn

**Affiliations:** 1 School of Applied Sciences, Huddersfield University, Huddersfield, United Kingdom; 2 Genomics, Synnovis, King’s College Hospital, London, United Kingdom

**Keywords:** coagulopathy, molecular pathology, haematology, haemoglobinopathy, molecular genetics

## Abstract

In almost all aspects of biomedical science, molecular pathology has brought unprecedented value in the diagnosis and management of human disease. Numerous commentators cite haematological disease as the leading genetic cause of global mortality and morbidity, and of these, those of the red blood cells are the most frequent. This narrative review, with a historical perspective, will discuss the role of genetics in these conditions, the leading pathology of red blood cells being the haemoglobinopathies, principally sickle cell disease and thalassaemia, with their many variants, and with potential roles for non-coding RNAs. The impact of genetics into conditions of the red cell cytoplasm will consider the enzymopathies, led by glucose-6-phosphate dehydrogenase deficiency, and extend to those of the cell membrane, causing disease such as hereditary elliptocytosis. Mutations in genes coding almost all the coagulation factors, and several platelet abnormalities, are discussed, as are those linked to conditions of iron overload. Previous, current and evolving technologies for diagnostic testing and their link with potential targeted therapeutic options for patient management are considered.

## Introduction

The development of molecular genetics and its clinical consequence of molecular pathology has been revolutionary, and haematology has been to the fore in the development of these disciplines. For example, Friedrich Miescher is credited with the first description of DNA, obtained as nuclein, from the nuclei of leukocytes [1], still the most useful source of genetic material. However, in the modern era, although several workers used the developing field of cytogenetics to investigate the aetiology of leukaemia [2], the “breakthrough” report of molecular pathology in haematology can be precisely mapped to that of Nowell and Hungerford demonstrating the karyotypic abnormality that came to be known as the Philadelphia chromosome [3]. Whilst the hereditary nature of the haemoglobinopathies had long been established, and its protein basis elucidated in the 1960s, it was in the 1970’s that molecular genetics demonstrated the precise chromosome sites of abnormalities that caused the disease, and then later the exact nature of the lesion at the level of the nucleotide strand, work that led to gene therapy [4–6]. This review aims to describe both a historical perspective and the current leading advances in the molecular pathology of non-malignant haematological disease.

### The Epidemiology of Haematological Disease

The Global Burden of Disease (GBD) study reported 55.9 million deaths in 2017, of which 834,700 were due to various forms of blood cancer and 104,600 due to haemoglobinopathies and haemolytic anaemias [7]. A separate report from 2016 reported a global prevalence of the major forms of blood cancer of almost 4 million people, a number dwarfed by the prevalence of all forms of haemoglobinopathy and haemolytic anaemia of around 2 billion people [8]. Thus, although the number of deaths due to blood cancer markedly exceeds that due to haemoglobinopathies and haemolytic anaemias by a factor of around 8, the prevalence of haemoglobinopathies and haemolytic anaemias exceeds that of blood cancer by some 500-fold.

These data, focussing on mortality, fails to address the morbid aspects of these conditions, which can be expressed as years lived with disability (YLD). The GBD report on this topic [8] told of some 460,000 YLDs in 2016 due to the major forms of blood cancer, data which is also dwarfed by the 6,572 million YLDs linked to haemoglobinopathies and haemolytic anaemias, an increase of some 14-fold, although that the lower YLD of blood cancer may be due to its higher mortality. Indeed, in 2011, the World Health Organisation estimated that around 7% of the world’s population to be carriers of a potentially pathological haemoglobin (Hb) gene, whilst more recently it has been estimated that 3 million people are living with sickle cell disease (SCD) alone, with over 300,000 affected births each year [9, 10]. But just as there are numerous variants of blood cancer and haemoglobinopathy, there are also many other haematological diseases with a genetic component, several with an impact into other forms of anaemia and haemostasis.

Of the hundreds of non-malignant haematological diseases, with the exception of a few, hard data on mortality and morbidity are scarce. The GBD study reported deaths in 2017 due to the most common of these: 7,200 to the thalassaemias, 38,400 to sickle cell disorders, 16,700 to glucose-6-phosphate dehydrogenase (G6PD) deficiency, and 42,200 to other haemoglobinopathies and haemolytic anaemias [6]. G6PD deficiency was recognised as a leading public health issue in India in 1966 [11]. The GBD also provides data regarding the prevalence and YLDs of these conditions (Table 1), which point to great variation in the extent to which each of the conditions bring disability. For example, crudely dividing the YLDs by the prevalence of each condition brings figure of between 0.01 to 0.08 for the haemoglobinopathies (smaller in each of the traits), but of the order or 1 × 10^−6^ for G6PD deficiency and 7.8 × 10^−5^ for its trait. This may be interpreted as a reduced impact of G6PD in terms of YLDs per case and so point to the haemoglobinopathies bringing most of the burden of disease in this setting.

**TABLE 1:** Global prevalence and years lived with disability of the haemoglobinopathies and haemolytic anaemias.

Condition	Prevalence	YLDs
Thalassaemia	366,000	23,000
Thalassaemia trait	287,107,000	3,260,000
Sickle cell disorders	3,888,000	317,000
Sickle cell trait	461,124,000	1,555,000
G6PD deficiency	331,513,000	26,000
G6PD trait	865,927,000	1,000
Other haemoglobinopathies and haemolytic anaemias	61,625,000	1,370,000

G6PD, glucose-6-phosphatase dehydrogenase; YLD, years lived with disability. From references [7, 8].

Perhaps the first reports of molecular genetics to dissect the genome with a view to molecular pathology was in the haemoglobinopathies, with the development of techniques such as the generation of globin gene cDNA from reticulocyte mRNA, and its subsequent insertion into bacterial plasmids [11–16]. The haemoglobinopathies may be classified with relative ease as the thalassaemias, the sickling disorders, and their hybrids.

## The Thalassaemias

### Molecular Genetics Unravelling the Basis of the Disease

During the 1960’s and early 1970’s, the use of Hb electrophoresis was the gold standard techniques for the study of abnormal globin chains, with advances in cell biology showing aberrant mRNA activity in thalassaemia. By mid-1970s the use of restriction nucleases and Southern blotting was consolidating the precise aetiology of the haemoglobinopathies at the level of the DNA [14–16]. These advances included reports of undetectable α-globin mRNA and deletions in the gene for α-globin as the cause of α-thalassaemia, and a case report of β -thalssaemia [17–19]. The latter is of historical value as it charts the use of specific techniques, such as the purification of reticulocyte mRNA by an oligo-thymine column, the use of this mRNA to generate cDNA using an RNA-dependent DNA-polymerase from the avian myeloblastosis virus, hybridisation of cDNA to ‘native’ DNA, and use of an *in vitro* cell-free translation model where globins are generated from purified mRNAs.

Of the several important proofs of concept were that, in contrast to normal mRNA, that from the affected patient generated α- and γ-globin but no β-globin, and their cDNA failed to anneal to normal β-globin DNA. A subsequent report identified three types of abnormal mRNA in beta°-thalassaemia–one with no detectable message, a second whose mRNA hybridised incompletely with cDNA, and a third that had intact mRNA that failed to translate, whilst another report pointed out that clinically severe and mild thalassaemias were linked to the deletion of both or a single α-globin gene, respectively [20, 21]. Orkin et al. used extensive restriction endonuclease digestion of DNA from patients with β^°^-thalassaemia, with fragments hybridised to β-globin cDNA, to demonstrate various deletions of the β-globin gene, some partial, some complete [22]. Other studies followed, showing a functioning ζ (zeta) gene in α-thalassaemia, and further studies of α-globin gene deletion [23, 24]. To these were added demonstration of the deletions of three of the four α-globin genes in HbH disease, and of all four genes in Hb Bart’s foetal hydrops, [25–27]. The latter, and others [28, 29] also examined the role of mRNA in these conditions, a process made relatively easy by harvesting mRNA from reticulocytes and other cells [13–15, 20]. Baralle and colleagues [30] used both Sanger and Maxam/Gilbert methods to sequence a 2,000-nucleotide section upstream of the ε-globin gene, a region now known to include a regulatory promotor region. Work of this nature was fundamental in that it subsequently pointed to a further genetic lesion, i.e., defects in a region outside an apparently normal functional gene (its promotor) could lead to a haemoglobinopathy [31].

A key point as regards many of these reports is that gene analysis was supported by protein electrophoresis, high-performance liquid chromatography (HPLC) and, in some cases, studies of the synthesis of globin chains, thus unequivocally defining the molecular basis of these diseases. A good example of this is the elucidation of the genetic basis of Hb Lepore, whose abnormal protein was demonstrated by starch block electrophoresis in 1958 [32], and whose genetic basis was confirmed 20 years later with the demonstration of a fusion of the β-globin gene and the δ-globin gene [33]. By the end of the 1970s, the mapping of the various isogenes of Hb, and their pathology, was becoming established and their locations defined: chromosome 16 for the α-globin gene locus (two copies: *HBA1* and *HBA2*) and chromosome 11 for the β-globin gene (*HBB*) and the γ-globin gene locus [12, 34–37], locations subsequently further defined as 16p13.3 and 11p15.4 respectively [38, 39].

A key methodological aspect of the work thus described was the used of section-specific restriction endonucleases to determine the presence or absence of particular sections of DNA and so produce a genetic map of a particular locus [40–42], but by the mid 1980s, nucleotide chain sequencing was becoming established. Sanger’s method was used to demonstrate the precise nucleotide sequence of a section of an intron in the β-globin gene of a G to T transversion [43], a T to G transversion [44] and an A to G transition [45], all causing a β-thalassemia. The Maxam and Gilbert method was used to report a case of α-thalassaemia with single base mutation in an α-globin gene at codon 116, that being GAG to UAG that formed a premature termination [46], whilst other part-sequenced the ε-globin gene [47]. We now describe these mutations as a single nucleotide polymorphism (SNP) [48].

A further initiative developed in the 1980s was the ability to use molecular genetics in the pre-natal diagnosis of hemoglobinopathies, initially using restriction endonucleases, then by lesion-specific oligonucleotide probes [49–54]. However, there remained a place for HPLC in determining the presence of abnormal Hbs in an antenatal setting [55–57]. At the end of the decade, Higgs and colleagues [58], and Cao and Murro [59], reviewed the genetics of the α-globin and β-globin gene loci respectively, summarising the various forms of abnormalities deletions, insertions, and SNPs causing different phenotypes of thalassaemia and other diseases of the α-gene and β-gene complex. Fuelled by work on transgenic mice, the 1990s saw molecular genetics used to identify the mechanism for the class switches of genes controlling the Hb isotypes from embryo to adult [60], the so-called “locus control region” (LCR). On the β-globin gene locus, this region is some 34 kB upstream (5′) of the ε gene [61], whilst others provided evidence of the involvement of the Erythroid Kruppel-like factor (subsequently re-named KLF-1, coded for by *KLF* at 19p13.13) in LRC regulation, binding a CACCC box in promotor regions, other important regions being a TATA box [62, 63]. A large deletion in a section of the LCR results in a form of gamma-delta-beta thalassaemia [64].

### Next-Generation Sequencing (NGS)

As technologies developed in the 2000’s, principally those of NGS analysis [65, 66], additional insights into the genetics of the thalassaemias were reported. These included α-gene duplication, defects in β-thalassaemia, α-gene quadruplication, enhanced prenatal diagnosis of β-thalassaemia, and population screening for both forms of thalassaemia [67–70]. A further advance was the ability to detect foetal nucleic acid in maternal blood, a technique used, alongside amplification refractory mutation system (ARMS) analysis and pre-implantation genetic diagnosis [71–74]. In Sardina, with a population of 1.7 million people, the carrier rate for β-thalassaemia is around 12%, such that 1 in 250 newborns is affected by this disease. Methods such as ARMS and other platforms found 25% of foetuses to be normal, 50% to be healthy carriers and 25% to be affected by the disease: over 98% of the latter pregnancies were terminated [75].

The ability to identify and quantify copy number variation (CNV) by quantitative RT-PCR can be improved by droplet digital PCR [76, 77], whilst whole-exome sequencing can be used to identify aberrant gene activity in thalassaemia [78, 79]. Described in 2010, third generation sequencing includes methods focussing on single-molecule sequencing technologies [80–82]. Examples of these methods include the Pac-Bio method, nanoball technology, and the Oxford Nanopore system, the latter used to detect rare and complex thalassaemia variants, and α-thalassaemia in preimplantation genetic testing [83–87]. These technologies have the ability to overcome the current shortcomings of short-read NGS for exon-level CNV of the globin genes, and provide a single test solution for combined analysis of single nucleotide variants and CNV.

### Gene Therapy for Thalassaemia

Whilst bone marrow transplantation is an established option for treatment, molecular pathology may provide the next step [4, 5, 88, 89]. The discovery and development of molecular genetics that came to be known as clustered regularly interspaced short palindromic repeats (CRISPR) [90] that, linked with Cas-9 endonuclease [91], has promised much, winning the 2020 Nobel Prize for Doudna and Charpentier with hope for therapeutic genome engineering [92]. One of the first practical uses of this technology was to correct a mutated β-globin gene in an induced pluripotent stem cell line *in vitro* [93], a process repeated by many others, giving proof of concept, with Sanger sequencing showing a corrected *HBB* [94]. Originally reported in respect of lymphoid malignancies [95], a genome-wide association study of 4,305 Sardinians (a population at high risk of haemoglobinopathy) [96], showed that *BCL11A* (coded at 2p16.1) is strongly linked to levels of HbF [97], and that this was linked to SNPs in a 3 kB section of the second intron [98]. These and other data proposed the hypothesis that manipulation of this gene, normally minimally active in the healthy adult, may lead to an increase in red cell HbF, a desired treatment for many haemoglobinopathies [96–98].

The revolutionary paper supporting this hypothesis, reported a patient with transfusion-dependent β-thalassaemia, and another with sickle cell disease who were ‘transplanted’ with autologous CRISPR-Cas9–edited CD34^+^ stem cells that were engineered to reactivate the production of foetal Hb by targeting *BCL11A* [99]. Over a year later, Hb levels had risen from 90 to 141 g/L, and from 72 to 120 g/L respectively, with parallel improvements in quality of life. A similar clinical study reported editing the *BCL11A* enhancer to increase Hb levels from 82 to 150 g/L, and from 108 to 140 g/L in two patients with transfusion-dependent β-thalassaemia [100]. Although a successful strategy, a meta-analysis suggested that the promotor regions of gamma globin genes *HBG1/2* may be a more efficient target than *BCL11A* [101]. Alternative uses of this technology include the silencing of genes for various red blood cell antigens, so creating red cells with considerable transfusion possibilities [102].

## The Sickling Disorders

As indicated in Table 1, the prevalence of SCD exceeds that of thalassaemia over 10-fold, and that of sickle cell trait (SCT) over thalassaemia trait by 61% [6, 7]. Data from 2008 points to the domination of sickle disorders in the 340,000 births globally with leading the list of Hb disorders (Table 2) [103, 104], although by 2021 this figure had risen to 515,000, with 7.74 million people living with the disease [105]. The leading hypothesis, postulated 70 years ago, for this very high level of an apparently pathological mutation is that it provides protection from infection by *Plasmodium falciparum* [106, 107].

**TABLE 2:** Breakdown of the proportions of births with a leading haemoglobinopathy.

Condition	%
Sickle cell anaemia	63.9
Sickle cell disease	16.1
β-thalassaemia major	6.8
HbE β-thalassaemia	5.6
Sickle/β-thalassaemia	3.3
HbH disease	2.8
HB Bart hydrops (α°/α°)	1.5

Data from refs [103–105].

### Molecular Genetics and the Basis of SCD

The precise lesions in the Hb molecules in the sickling disorders has long been known, and the electrophoresis technique developed in the 1950s was extended to further refine knowledge of the amino acid nature of the sickling condition during the 1960s [108–110]. In the 1970s this was extended to the different globin species and their roles in various haemoglobinopathies [111], enabling putative maps of the globin amino-acid chains [112]. As with the thalassaemias [17–20], the 1970s also saw the genesis of the molecular pathology of sickling disorders, primarily with restriction endonucleases [113–115]. Perhaps the first major use of molecular genetics was made by Marotta and colleagues [116] in the definition of the lesion that causes SCD. They purified mRNA from sickle cell reticulocytes using an oligo(dt)-column, and from this used avian myeloblastosis virus RNA-dependent DNA polymerase to generate cDNA that was subsequently phosphorylated. Restriction endonucleases digested this cDNA, and fragments were sequenced by the method of Maxam and Gilbert, showing the HbS to be due to the result of a single nucleotide base mutation in the β-globin gene that converts the glutamic acid codon (GAG) at amino acid position 6 to one for valine (GTG) [117]. A further advance being the detection of β-globin fragments in cells from amniotic fluid that were consistent with sickle-cell trait [118, 119].

As the 1980s proceeded, the work with the use of restriction endonucleases regarding achieving gene maps of the α-globin and β-globin loci [33, 58, 120, 121] was being confirmed and extended by direct genetic analysis, with developments such as isotopic and non-isotopic probes, and their use (alongside PCR) in prenatal diagnosis [122–125]. Subsequent reports described *in situ* dot hybridisation, amplification by PCR of a 725 bp section of the β-globin gene with the sickle-cell anaemia point mutation in formalin-fixed bone marrow samples [126, 127], and allele-specific PCR, used to analyse the lesion in HbC [128, 129]. A 1992 review summarised the potential for using molecular genetics to insert functioning α-globin and β-globin genes into haemopoietic stem cells via a viral vector [130]. This period also saw the development of PCR heteroduplex analysis, used to screen for HbS and HbC, and the use of amplification refractory mutation system to detect HbA/HbS chimerism after bone marrow transplantation for sickle cell anaemia [131–133].

### Molecular Genetics Meets Cell Biology

Developments in cell biology and transfection were used in numerous studies of the basic biology of the sickle phenotype and genotype, with prototype gene therapy. Tang et al. [134] showed that the promoter sequences of the δ-globin gene differs from the β-globin gene by the absence of an erythroid Krüppel-like factor (EKLF) binding site and alteration of the CCAAT box to CCACC, and that restoration of this site can increase δ-globin gene expression, a strategy that has potential future clinical benefit. Knowledge of the sequence of the promotor region of the γ-globin gene enabled Graslund and colleagues [135] to insert an artificial transcription factor for this gene into a cell line, which resulted in an increased expression of α_2_γ_2_ HbF, and so a strategy for increasing levels of this Hb variant in SCD, a leading therapeutic option [136]. Ho and colleagues [137] generated recombinant mutants of sickle Hb in *E coli* to show that the formation of HbS polymerisation requires interaction between α-globin 114Pro and β-globin 87THr, and that a mutant 114Arg inhibits the polymerisation, whilst Cole-Strauss et al. [138] inserted a chimeric DNA/RNA to lymphoblastoid cells in a direct correction of the mutation in the Hb betaS allele.

A step closer to clinical application was taken by Pawlik and colleagues [139], who corrected SCD in a mouse model by inserting a lentivirus vector carrying a beta globin gene variant that prevents HbS polymerisation into haemopoietic stems cells, which was then passed into the mouse. They subsequently transduced human cord blood cells with the vector, and transplanted them into mice, using PCR to demonstrate integration of the human gene, with increased levels of the modified β-globin [140, 141]. Small-interfering RNA represents a new opportunity to treat various diseases [142], an example being use of a small interfering antisense RNA to silence a β^s^-globin gene when transfected into HeLa cells and an erythroleukaemic cell line [143]. Vasavda and colleagues [144] used RTqPCT to measure circulating *HBB* DNA in the plasma of patients with SCD, HbS/β^o^ thalassaemia, HbSC and HbAA controls, finding no difference in steady state disease, but increased levels (alongside CRP and the white blood cell count) when in crisis.

A long-addressed strategy in SCD is to increase levels of HbF, and so ameliorate the clinical effects of the mutation, as does hydroxyurea, a drug that reduces levels of plasma *HBB* [145]. Using a variety of techniques that included mass spectrometry, RT- PCR and a custom-made Illumina platform, Sebastiani and colleagues [146] reported links between HbF and SNPs in *TOX* at 8q21.1, in *GPM6B* at Xp22.2, and at 15q22 (a region that includes *AQP9, MAP2K1, SMAD3,* and *SMAD6*), suggesting manipulation of these gene may influence levels of HbF. As in thalassaemia [99–101], *BCL11A* may be important in SCD. Zhou et al. [147] showed that knockdown of KLF1 in adult erythroid stem cells markedly reduces *BLC11A* levels and increases the ratio of γ-globin to β-globin. Uda et al. [96] used genome-wide association studies to show that *BCL11A* is associated with HbF levels in sickle cell patients (as it is in β-thalassaemia), whilst Ghedira and colleagues [148] used quantitative multiplex PCR to estimate that the loss of the BCL11A binding domain leads to an increase in HbF of 27 g/L. Chen and colleagues [149] used an electrophoretic mobility shift assay and other methods to show links between a SNP in a GATA-1 binding motif and high levels of HbF. Other genes linked to HbF include *OR51B5* and *OR51B6*, both coded at 11p15.4, *HBS1L-MYB* at 6q23, and *SAR1A* at 10q22.1 [150–152].

### Gene Therapy for SCD

Both these strategies came to clinical fruition in the decade that followed, fuelled by the slow and steady increase in knowledge of practicalities of gene therapy, such as inserting genes into cells [153–160] (Table 3). A comparative study concluded that *BCL11A* is the most clinically relevant approach for CRISR/Cas9 insertions into stem cells [161]. In 2017, Ribeil and colleagues [162] published a case report of a thirteen-year-old boy with homozygous SCD who was transplanted with his own CD34^+^ stem cells that had been engineered with a Lentivirus vector to carry the *HBB* variant β^A-T87Q^, a molecule that inhibits the sickling process. He was discharged on day 50, and after 15 months, the patient’s total Hb increased from 101 to 118 g/L with a fall in reticulocytes from 238,000 to 141,000 per mm^3^, with no recurrence of pathological sickle crises. In 2021, Esrick and colleagues [163] reported six patients with SCD who were transplanted with autologous CD34^+^ stem cells transduced with a lentivirus vector carrying a short-hairpin RNA targeting *BCL11A*. HbF induction was robust and stable, with all indices of HbF improved (e.g., HbF rising from 9.0 to 18.6 pg/cell) with a reduction or absence of clinical manifestation of the disease.

**TABLE 3:** Methods for introducing genes into cells.

Method	Example
Lentivirus vector	An engineered *HBB* designed to impede red cell sickling into human CD34^+^ bone marrow cells, the resulting red cells exhibiting less sickling upon deoxygenation [153]
Lentivirus vector	Use of a small hairpin RNA to knockdown *BCL11A* in normal and sickle cell CD34^+^ cells generating red cells with γ-chain expression and so increased HbF [154]
Sleeping beauty transposon	An anti-sickling gene into CD34^+^ from a sickle cell patient which produced red cells with a less pathological phenotype [155]
Tal-effector nuclease	A full-length β-globin gene inserted into K562 (a myeloid leukaemia cell line) [156]
CRISPR/Cas9	Correction of SCD patients bone marrow CD34^+^ cells and production of wild-type haemoglobin A [157]
CRISPR/Cas9	Deletion of a 13 kB section of the β-globin locus in normal CD34^+^ stem cells, thus mimicking HPFH, resulting in increased expression of γ-globin gene expression [158,159]
CRISPR/Cas9 and adenovirus vector	Correction of the mutation responsible for SCD in patient-derived stem and progenitor cells with expression of adult β-globin (HbA) messenger RNA in erythrocytes [160]

Figures in parentheses are references.

As descrived previously, the same transplant strategy was used by Frangoul et al. [99] to treat a 19-year-old female with the β^o^/β+ thalassaemia but also a 33-year-old female with SCD (βS/βS and a single α-globin deletion). This resulted in increases in total Hb from 90 g/L to 142 g/L after 15 months, and from 72 to 120 g/L, whilst the % of red cell expressing HbF increased from 10.1 to 100, and from 33.9% to 98.1%, respectively. Vaso-occlusive events were eliminated in the patient with SCD. The following year, Kanter and colleagues [164] reported the use of a lentivirus vector to insert a gene coding an antisickling engineered variant of HbA into autologous stem cells, subsequently transplanted back into the patient with SCD. The largest study so far (n = 35) of this engineered Hb saw total Hb rise from a median of 85 g/L to over 120 g/L (53% being the engineered Hb) after 36 months, with no severe vaso-occlusive events after transplantation. Levels of serum lactate dehydrogenase and serum indirect bilirubin, and the reticulocyte count, all fell markedly. Magrin and colleagues [165] reported a 36, 42, and 60 months follow up of three SCD patients with the antisickling Hb variant β^A-T87Q^, noting marked variation in the % of total Hb being composed of HbF and HbA^A-T87Q^ of 43.7%, 14.0% and 51.1% respectively. Sharma et al. infused stem cells bearing a CRISPR-Cas9 product engineered to disrupt *HBG1* and *HBG2* into three patients with severe SCD [166]. This resulted in sustained induction of red cell foetal Hb, that being 19%–26.8% of total Hb, with 69.7%–87.8% of erythrocytes classified as F cells, and a decrease in the manifestations of SCD. Extending their previous work [163], Esrick and colleagues now [167] compared their patients transplanted with *BCL11A*-silenced stem cells with transplanted patients being treated with standard care hydroxyurea. The % of red cells from transplanted patients containing ≥10 pg of HbF was comparable to that of hydroxyurea high-responders, but superior to that of hydroxyurea low-responders, but the % of cells carrying HbS was lower than both hydroxyurea groups.

Whilst gene therapy represents an alternative to bone marrow transplantation, which although well-established, has its drawbacks [168], the number of treated patients is small and follow-up short, such that full assurance is as yet unforthcoming. Indeed, despite the apparent success of some cases where all three patients in the OTq923 study (targeting BCL11A) [166] showed a clinical improvement, they continued to have mild haemolysis and some symptoms of sickle-cell disease, a finding which may have contributed to the decision by the sponsor to discontinue the development of this product [169]. The treatment cost of $2 million may be balanced by reduced cost of, for example, vaso-occlusive events, acute chest pathology etc. over the hoped-for decades of life remaining and the inestimable cost of improved quality of life [170]. Chapman et al. have pointed out the danger of vector insertion related leukaemias in other diseases from 2003 to 2014 and found an increased frequency of potential driver mutations associated with myeloid neoplasms or clonal haematopoiesis in both genetically modified and unmodified stems cells after SCD transplantation [171]. It remains to be seen if these mutations precipitate other disease and prompts the possible need to screen for these mutations before gene therapy is commenced.

## Concurrent Thalassaemia and Sickling Disorders

Electrophoresis and other methods have demonstrated the co-inheritance of different variants of the hemoglobinopathies, largely concurrent thalassaemia and SCD, but also of HbC with α-thalassaemia, and HbC with HPFH ([172–176]). These have subsequently been confirmed by techniques such as allele-specific PCR and restriction endonuclease mapping [177–180]. Kundrapu and colleagues used Sanger sequencing to define a compound heterogeneity in Hb D-Ibadan and HbC [181], whilst Wilcox and colleagues confirmed the genetic basis of Hb Kenya as being due to non-homologous crossing-over of β-globin and γ-globin genes, resulting in a fusion protein [182], and Redding-Lallinger et al. used NGS to define the gene lesions in HbS/HbD-Ibadan and β^+^thalassaemia/Hb D-Ibadan [183]. Notably, the genetics distinguished the Ibadan variant of HbD from the Los Angles variant, a feat not possible with electrophoresis.

## Non-Coding RNAs (ncRNAs) in the Haemoglobinopathies

Whilst ncRNAs include ribosomal and transfer RNAs, these molecules are taken to include two major groups, the long non-coding RNAs and the microRNAs known to have regulatory roles in a number of diseases [184]. One of the first examples in the haemoglobinopathy setting involved insertion of a lentivirus vector carrying a small nuclear RNA into stem cells from a patient with β-thalassaemia, resulting in the correction of an aberrant splice site and increased levels of HbA [185]. Chen and colleagues, using RT-PCR and micro-array analysis, reported a large number of miRNAs in reticulocytes and red cells: mean miRNA expression was markedly higher in HbSS reticulocytes, whilst miR-320 expression was markedly higher in HbAA erythrocytes, whilst low levels in HbSS cells were linked to defective CD71 downregulation [186]. Notably, in view of the interest in *BCL11A* as a target for gene therapy [96–101, 161, 162, 166], miR-486-3p and miR-210 are regulators of this gene, with over-expression resulting in *BCL11A* suppression and so increased expression of the γ-globin gene [187, 188]. Others used RT-PCR to determine plasma levels of miR-451 and miR-155, finding increased expression in β^°^-thalassaemia/HbE, and a link between miR-451 in severe disease and reticulocyte counts [189]. Lai et al. [190] used a reticulocyte RNA microarray analysis in patients with HPFH and β-thalassaemia, reporting that 862 lncRNAs (605 upregulated, 257 downregulated) and 568 mRNAs (324 upregulated, 244 downregulated) showed a >2-fold change. Although much development work is needed, several commentators have hypothesised uses for ncRNAs as therapeutic options [191, 192].

## The Enzymopathies

Whilst there are numerous mutations in genes coding for enzymes and other forms of haemolytic anaemia (Table 1), such as those linked to abnormal components of the erythrocyte membrane, the leading non-haemoglobinopathy genetic disease is glucose-6-phosphate dehydrogenase (G6PD) deficiency [5, 7, 10]. This disease is often cited as the most common enzymopathy, which in 1996 affected some 400 million people globally [193], and 20 years later had risen to almost 1.2 billion [7, 8]. Despite this figure, the number of deaths due to this disease is roughly twice that of the thalassaemias, but half that of the sickling conditions, due mainly to the more benign course of the disease and the variability and penetrance of the many mutations of its associated gene at Xq28.

As with the haemoglobinopathies, the leading driver of the deficiency is protection from malaria [194, 195]. A report from India in 1966 indicated a biochemically defined frequency of 3.6% in males and 7.7% in females in a population from Bengal, 7.4% in males and 31.0% in females in a population from Bombay, and 4.6% in a population from Calcutta unstratified for sex [10]. A later review described a global prevalence ranging from 2.9% in the Pacific region, 3.9% in Europe, to 6.0% in Africa, although these data depend on the method of assessment, which ranges from 3.6% for the NADPH fluorescence method to 6% for DNA analysis [196]. In Arab countries, prevalence ranges from 2% to 31% [197], and in countries targeting malaria elimination, an allele frequency of 5.3% has been reported [198].

From a partial amino acid sequence of the enzyme, Persico et al. synthesised a 17-mer probe from which to screen a cDNA library prepared from partially purified G6PD mRNA, with sections sequenced by the methods of Maxam and Gilbert, and of Sanger [199]. From this, the same group published further details of the 18 kB gene and its 13 exons and subsequently used Sanger sequencing of λ-phage cDNA clones to report SNPs in seven variants whose red cell enzyme activity ranged from <2% to 84% of normal levels [200]. Concurrently, Takizawa and colleagues purified G6PD to homogeneity, derived its 531 amino acid sequence, and from this constructed a 41-mer synthetic sequence subsequently labelled with P^32^ [201]. They then constructed bacteriophage vector cDNA libraries using mRNA from hepatocytes and a hepatoma cell line, which they transfected into *E coli*, screened selected colonies by hybridising with the 41-mer probe, and sequenced selected fragments by Sanger’s method. Vulliamy et al. [202] used a similar phage vector library strategy to demonstrate SNPs in *G6PD* from patients with G6PD deficiency that would lead to an abnormal product and so the haemolytic anaemia typical of the disease. Thus, whilst the molecular pathology of G6PD deficiency lags somewhat behind that of the haemoglobinopathies, the genetic basis of the disease has been elucidated [193, 203].

The second most important enzymopathy, the autosomal recessive pyruvate kinase deficiency (PKD), is caused by loss-of-function mutations in the coding gene *PKLR* at 1q22, and which is used in diagnosis and population screening [204]. With a frequency of some 1/20,000, the >300 mutations lead to viable clinical pictures, and overall PKD brings a 10-year reduction in lifespan with a hazard ratio (95% confidence interval) of 5.0 (1.9–13.4) [205]. Although there are examples of successful bone marrow transplantation [206], molecular therapies are as yet unreported.

## Haemostasis

This section is easily divisible into pathology of the platelet and of coagulation factors, the latter being dominated in a clinical setting by haemophilia.

### Haemophilia

Perhaps the oldest known genetic disease, as described in ancient Hebrew texts, haemophilia is the likely reason for the dispensation of the surgical removal of the foreskin (circumcision) in a neonate if older brothers died following prolonged bleeding after their own circumcision. In the modern era, the disease was clearly described over 200 years ago [207], although not named, and perhaps the oldest detailed clinical description of haemophilia is over 150 years ago [208], with a treatment of the infusion of serum at the beginning of the last century [209].

The gene for Factor VIII (*F8*) was cloned, sequenced (reporting 186 kB, 25 introns, and 26 exons) and its 2,351 amino acids (giving a mass of 267-kDa) determined in 1984 [210–213]. Shortly afterwards, FVIII-specific probes were used for the prenatal diagnosis of haemophilia, and its chromosomal location determined [214–217]. By the end of the decade, deletions, SNPs, inversions, and insertions in the gene had all been shown to be the cause of haemophilia [218–222], reviewed in [223]. Interestingly, a cluster of mutations, many of which are inversions, in intron 22 found in 40% of one series of haemophiliacs can cause defective joining of exons 22 and 23 in the mRNA and so result in the disease [224].

The closely-related Christmas disease was described in 1952 [225], the identity of the restorative agent (anti-haemophilic factor) was determined in the late 1950s as Factor VIII. Becoming known as haemophilia B to distinguish it from ‘standard’ haemophilia, subsequently known as haemophilia A, the gene coding Factor IX (i.e., *FIX*) was cloned in 1982, defects shown in 1985 to be the cause of deficiency of the protein [226–228]. Kurachi and Davie [229] used a baboon liver mRNA to synthesise a radiolabelled cDNA 14-mer primer to identify and clone sections of a human liver cDNA library that were inserted into plasmids. Restriction endonuclease digestion of positive clones provided fragments that were sequenced by the method of Maxam and Gilbert. The gene has eight exons transcribing a 2.8 kB RNA that translates to a pre- and pro-peptide and mature protein of 415 amino acids, subsequently cleaved to FIXa with a relative molecular mass of 57 kDa. Standard sequencing reported 55.8% of 1692 abnormalities to be point mutations, 20.8% to be polymorphisms,16.6% to be deletions, and 83.9% to be in exons, the most common being in the eighth (37%) and second (12%) [230]. The power of NGS was demonstrated by the analysis of DNA from 2401 patients with haemophilia A and 599 with haemophilia B, finding 924 unique variants, confirming the inversion in intron 22 to be the leading mutation in severe cases of haemophilia A (42% of cases), followed by frameshift and missense (both 17.4%), whilst missense mutation were the most common in mild and moderate disease (79.5%) [231]. In haemophilia B, the leading mutation was missense in both severe (47.2%) and mild/moderate (87.1%) disease.

The relatively small size of *F9* (∼34 kb), normally coding a 2.8 kb mRNA, permits its insertion into a viral vector that can be delivered to hepatocytes that then produce the active zymogen product of some 461 amino acid residues [232]. Furthermore, molecular genetics has provided a further advance in identifying a mutated form (*FIX-Padua*) that generates a F9 with enhanced enzyme activity compared to normal F9, and which is effective in a clinical setting [233]. This part-contrast with *F8*, of 186 kb, coding an mRNA of 9 kb and ultimately a 2,300 amino acid product, that together provided a challenge for genetic engineers [234]. However, these have been overcome, and two therapeutics (Valoctocogene Roxaparvovec and Efanesoctocog Alfa) are now available for the treatment of haemophilia A [235].

### Other Coagulation Factors

By 1971 the hereditary nature of dysfibrinogenaemia (as autosomal dominant), afibrinogenemia, hypoprothrombinaemia, dysprothrombinaemia, defects of Stuart-Prower factor (Factor X) (all autosomal recessive) had been described, all pointing to a genetic lesion [236]. The molecular pathology of all other factor deficiencies was subsequently defined, locations of leading coagulation genes, and their population frequencies, are shown in Table 4.

**TABLE 4:** Coagulation factor genes.

Gene	Location	Frequency of deficiency per million
*F2*	11p11.2	0.5
*F5*	1q24.2	1
*F7*	13q34	2
*F8*	Xq28	200^a^
*F9*	Xq27.1	33^a^
*F10*	13q34	1
*F11*	4q35.2	1
*F12*	5q35.3	1
*F13*	6p25.1 and 1q31.3	0.5
*vWf*	12p13.31	1^b^

^a^ Males.

^b^ The most severe phenotype. Factor XIII is composed to two protein chains.

Based on an amino acid sequence of a factor Va light chain, Jenny and colleagues [237] synthesised a 39-mer probe and used it to screen an oligo(dT)-primed human foetal liver library, with clones expressed in phage vectors. Overlapping fragments were sequenced by Sanger technology, reporting a 6672 bp coding region ultimately producing a mature protein of 330 kDa consisting of 2196 amino acids. Subsequent analysis showed the presence of 25 exons, and that removal of a domain from the protein structure generates FVa, formed from a 105 kDa heavy chain and a 71 or 74 kDa light chain [238]. The combined deficiency of FV and FVIII may follow loss-of-function mutations (mostly insertion/deletions and splice site mutations) in *LMAN1* at 18q21.32, and *MCFD2* at 2p21 that together code for membrane proteins of the endoplasmic reticulum and Golgi apparatus pathway for transporting the coagulation factors [239].

The gene for FVII is located 2.8 kB telomerically to that for FX, spanning 12 kB and with nine exons coding for a mature protein of 406 amino acids of 50 kDa, the leading cause of deficiency being a 10 bp insertion polymorphism, with other abnormalities linked to a variable reduction in biological activity [240]. Fibrinogen is formed from three proteins, coded for by *FGA* (7.6 kB, 6 exons), *FGB* (8 kB, 8 exons)*,* both at 4q31.3, and *FBG* (8.5 kB, 10 exons) at 4q32.1, giving a total mass of 340 kDa: the estimated prevalence of deficiency (generally a deletion, most often in *FGA*) is around 1/million. Vitamin K dependent coagulation factor deficiency, producing low levels of several of the products of these genes, is linked to *GGXC* at 2p11.2, coding for gamma-glutamyl carboxylase, and *VKORC1* at 16p11.2, coding for a subunit of the vitamin K epoxide reductase complex [241–243].

The most common genetic haemorrhagic disease is von Willebrand disease (VWD), with an estimated Caucasian population frequency of 0.57%–1.15%, translating to 5,700 to 11,500 per million [244], far exceeding that of haemophilia A at 200 per million males. However, clinical VWD runs a spectrum from asymptomatic to life-threatening, where the phenotype resembles haemophilia, such that the referral rate for the most severe cases has been estimated at 23 to 113 per million [245]. The variable nature of the disease, characterised by low levels of von Willebrand factor (vWf), can be accounted for by various mutations in *VWF* at 12p13.31, where it spans 176 kB, consisting of 52 exons between 52 base pairs and 1.3 kB generating an mRNA around 9 kB long [246, 247].

In contrast to low levels of plasma vWf, increased levels are common in cardiovascular disease, cancer, and in connective tissue diseases, reflecting an endothelial cell pathology, although the driver(s) are unlikely to be genetic [248]. Nevertheless, a leading feature of this molecule is as a chaperone for FVIII, increased levels of which are a risk for a venous thrombosis, with both molecules considered by some to be part of a thrombophilia screening panel [249], although this view is not universal [250]. Genes cited as leading genetic causes of thrombophilia are shown in Table 5, many of which may be part of a multi-target NGS thrombophilia panel for investigating those with, or at risk of, a venous thrombosis [250–255], as summarised in a 2022 guideline [256].

**TABLE 5:** Genetics of leading forms of thrombophilia.

Gene	Product	Location	Pathophysiology
*SERPINC1*	Antithrombin	1q25.1	Deletion and loss of function SNPs lead to qualitative and quantitative deficiency
*FV* rs6025	Factor V Leiden G1691A	1q24.2	Missense SNP generates a FV resistant to Protein C
*F2* rs1799963	Prothrombin G20210A	11p11.2	SNP in the 3′ untranslated region leads to increased transcription
*PROC*	Protein C	2q14.3	Deficiency leads to failure to inactivate FVa and FVIIIa
*PROS1*	Protein S	3q11.1	Obligate co-factor required for Protein C function

### Platelet Abnormalities

Nurden and Nurden [257] summarised the genetics of the ontogeny of thrombopoiesis in four stages:

- *THPO* at 3q27.1, coding for thrombopoietin, and *MPL* at 1p34.2 coding the thrombopoietin receptor (loss of function mutations in either causing congenital amegakaryoctic thrombocytopenia), at the level of the haemopoietic stem cell.
- Subsequent roles for *HOXA11* at 7p15.2*, FLI1* at 11q24.3, *RUNX1* at 21q22.2, *GATA1* at Xp11.23*, GFI1B* at 9q34.13, and *ETV6* at 12p13.2, all genes coding for a transcription factor, as the megakaryoblast develops.
- As the megakaryocyte develops, *MYH9* at 22q12.3, coding myosin heavy chain 9*, DIAPH1* at 5q31.3, coding diaphanous-related formin, a molecule involved in actin polymerisation*, FLNA* at Xq28, coding filamin, also with a role in actin structure, and *TUBB1* at 20q13.32, coding tubulin, contributing to cytoskeletal integrity, become relevant.
- The final stage, that of proplatelet development, involves *VWF*, *ITGA2B* (at 17q21.31, coding the integrin CD41/gpIIb)*, ITGB3* (at 17q21.32, coding CD61/gpIIIa, also an integrin), *GP1BA* (at 17p13.2, coding CD42b/gpIb-α)*, GP1BB* (at 22q11.21, coding CD42c/gpIb-β)*,* and *GP9* (at 3q21.3, coding CD42a/gpIX).

The leading forms of genetic disease of platelets involve surface receptors. In Bernard Soulier syndrome (with a prevalence of 1 per million), there is a thrombocytopenia and a qualitative change, the latter caused by failure of any of *GP1BA* (the most frequently mutated gene)*, GP1BB, GP9*, or *GP5* (at 3q29) to correctly code molecules forming the GpIb-IX-V complex, the receptor for vWf. Failure of this receptor/ligand reaction results in platelets unable to form a thrombus, and so haemorrhage [258, 259]. Glanzmann thrombasthenia also has an incidence of 1/million, but in areas of high consanguinity this may increase five-fold. It is caused by mutations in *ITGA2B* or *ITGB3*, that together code for integrins GPIIb/IIIa, also known as the fibrinogen receptor [260]. The molecular pathology of the platelet extends other forms of haemorrhagic disease (Table 6) [257, 261–265]. Gebetsberger and colleagues [266] summarised Sanger, NGS panels, WES and WGS approaches to platelet disorders.

**TABLE 6:** Genetics of leading platelet disorders.

Gene	Product	Location	Pathophysiology
*RBM8A*	An RNA binding protein	1q21.1	Thrombocytopenia with absent radius (the forearm bone)
*SBDS*	A ribosome maturation factor	7q11	A thrombocytopenia in Schwachman-Diamond syndrome
*MYH9*	Myosin heavy chain	22q12.3	Thrombocytopenia and large platelets, and present as part of the May-Hegglin anomaly, and the syndromes of Sebastian, Epstein and Fechtner
*WAS*	Multiple roles, including actin polymerisation	Xp11.23	Wiskott-Aldrich syndrome, with thrombocytopenia, small platelets, immunodeficiency And eczema
*NBEAL2*	Scaffolding protein in alpha granules	3p21.31	Grey platelet syndrome: thrombocytopenia

## The Red Blood Cell Membrane and Cytoskeleton

As with the haemoglobinopathies, the geographical distribution of some abnormalities of the erythrocyte membrane and cytoskeleton support the hypothesis that they have developed in response to malaria. Autosomal dominant hereditary elliptocytosis (with an incidence of 0.6%–3% in West Africa) results from mutations in *SPTA1* at 1q23.1 or *SPTB* at 14q23.3, coding for α- or β-spectrin (together, 20% of cases), *SLC4A1* at 17q21.31, coding for band 3, the chloride/bicarbonate anion exchanger (15%–20%), or in *EPB41* at 1p35.3, coding for another cytoskeletal component, band 4.1 (<15% of cases) [267]. Autosomal recessive hereditary pyropoikilocytosis (also frequently diagnosed in infants of African ancestry) may also be linked to defects in *SPTA1* or *SPTB*, whilst autosomal dominant Southeast Asian ovalocytosis (principally found in Malaysia, Thailand, Indonesia, the Philippines and Papua New Guinea) is caused by a 27-nucleotide deletion in *SLC4A1*, which confers a resistance to cerebral malaria, with a mean allelic population frequency of ≤1% [268].

Hereditary spherocytosis is the most common hereditary haemolytic anaemia worldwide, and in Caucasians has a prevalence of 0.02%–0.04%. with some 75% being autosomal dominant, although the frequency in China is estimated at 0.014%. The most common causative lesions are in *ANK1* at 8p11.21 (e.g., c.-108T>C, c.-153G>A, and c.-204C>G) coding for ankyrin (50%–60% of cases), followed by *SLC4A1*, *SPTA1, SPTB*, and *EPB42* at 15q15.2, coding for protein 4.2 [269, 270].

Hereditary stomatocytosis is an autosomal-dominant condition where the transport of cations (notably sodium and potassium) is impaired, leading to osmotic misbalance, resulting in two abnormal forms. An overhydrated form is linked to missense variants of *RHAG* at 6p12.2, coding for Rh-associated glycoprotein (CD241), which functions as an ammonia transporter, whilst over 50% of a dehydrated variant is due to one of three mutations in *PIEZO1*, at 16q24.2, coding a mechanosensitive cation channel [271, 272]. Another, the Gardos channelopathy, is associated with *KCNN4* at 19q13.31, coding a calcium-activated potassium channel. A mild form of stomatocytosis, cryohydrocytosis (present at temperatures <20 °C), results from a missense mutation in *SLC4A1* [270, 272].

## Iron Overload

The leading disease in this group is hereditary haemochromatosis, the most common such genetic condition in those of European descent, present in its homozygous form in 0.2%–0.33%, with some 9% of the population being heterozygotes. There are several variants. In Type 1, causing 75%–80% of cases, the lesion is in *HFE* (of 7 exons, and 12 kB long, located within the major histocompatibility complex) at 6p21.3-22.2, with 96% of these being at position 845, generating p.C282Y/p.Cyst282Tyr, and 4% being the p.C282Y/p.Hist63Asp (p.H63D) compound heterozygote genotype. *HFE* codes for a mature 363 amino product that binds to the transferrin receptor 1 and is also a co-factor for hepcidin synthesis, where loss of function leads to reduced hepcidin mRNA, decreased plasma hepcidin, and so excessive tissue iron accumulation [273, 274].

Type 2a disease is caused by variants of *HJV* (or *HFE2*) at 1q21, coding for a molecule linked to the membrane receptor for bone morphogenetic protein (BMP), whilst type 2b concerns *HAMP* at 19q31, coding for the primary iron regulator molecule hepcidin. BMP6 itself, coded for by *BMP6* at 6p24, is a further component of the regulation of hepcidin. Type 3 disease follows from a mutation in *TFR2* at 7q22.1, and of 20 kB length, the most common being C>G in exon 6, creating a nonsense mutation of p.Y250X. A case report described a second mutation in exon 2, producing nonsense mutation of p.E60X, both mutations resulting in loss of transferrin receptor 2 [275].

The lesion in type 4 disease (also described as ferroportin disease) is within *SCL11A2* at 12q13.12, which codes for ferroportin (FPN), a cell membrane component that regulates the passage of iron from the enterocyte to the plasma, and which itself is regulated by hepcidin. Mutations such as an exon 3 SNP resulting in alanine to aspartic acid at residue 77 (p.A77D), result in an FPN unable to export iron, which therefore accumulates inside the cell. A second from of ferroportin disease is characterised by a *SCL11A2* mutation, resulting in a variant of ferroportin that is resistant to regulation by hepcidin and so exports excessive amounts of iron [276–278]. A genome-wide meta-analysis confirmed the importance of *HFE*, *HAMP* and *SLC11A2* but also reported a possible role for *TMPRSS6* at 22q12.3, coding for transmembrane serine protease 6 [279]. Other data from this study pointed to significant odds ratios (95% confidence) for the rs748587164 SNP A/T minor/major allele in *TF* at 3q22.1, coding for transferrin, of 3.89 (2.46–6.15, p = 5.8 × 10^−9^), the rs1800562 SNP A/G in *HFE* of 3.54 (3.29–3.8, p = 2.2 × 10^−255^), rs1799945 SNP G/! in *HFE* of 1.44 (1.34–1.55, p = 1.3 × 10^−24^), and the rs855791 SNP A/G in *TMPRSS6* of 0.75 (0.71–0.79, p = 1.9 × 10^−26^).

Although involved in copper metabolism, autosomal recessive variants in *CP* at 3q24-25.1, coding for caeruloplasmin, and leading to acaeruloplasminaemia, may also lead to iron overload, almost certainly linked to its ferroxidase activity [280].

## Conclusion

Molecular pathology has without doubt provided a revolution is our basic understanding of aetiology, diagnosis and management of numerous diseases, including those of the red blood cell, the haemostasis system, in iron metabolism, and also in blood cancer [281]. One may speculate that genetic testing will continue to be an important and expanding feature of clinical and biomedical science, and conceivably may develop into near-patient testing in primary care and other settings, perhaps even into self-testing. In doing so, disease may be recognised earlier and so addressed with more focus, ideally reducing the need for more complex healthcare referrals.
